# Estrogen and androgen-converting enzymes 17β-hydroxysteroid dehydrogenase and their involvement in cancer: with a special focus on 17β-hydroxysteroid dehydrogenase type 1, 2, and breast cancer

**DOI:** 10.18632/oncotarget.15547

**Published:** 2017-02-20

**Authors:** Erik Hilborn, Olle Stål, Agneta Jansson

**Affiliations:** ^1^ Department of Clinical and Experimental Medicine and Department of Oncology, Faculty of Health Sciences, Linköping University, Linköping, Sweden

**Keywords:** breast cancer, estrogens, androgens, HSD17B1, HSD17B2

## Abstract

Sex steroid hormones such as estrogens and androgens are involved in the development and differentiation of the breast tissue. The activity and concentration of sex steroids is determined by the availability from the circulation, and on local conversion. This conversion is primarily mediated by aromatase, steroid sulfatase, and 17β-hydroxysteroid dehydrogenases. In postmenopausal women, this is the primary source of estrogens in the breast. Up to 70-80% of all breast cancers express the estrogen receptor-α, responsible for promoting the growth of the tissue. Further, 60-80% express the androgen receptor, which has been shown to have tissue protective effects in estrogen receptor positive breast cancer, and a more ambiguous response in estrogen receptor negative breast cancers. In this review, we summarize the function and clinical relevance in cancer for 17β-hydroxysteroid dehydrogenases 1, which facilitates the reduction of estrone to estradiol, dehydroepiandrosterone to androstendiol and dihydrotestosterone to 3α- and 3β-diol as well as 17β-hydroxysteroid dehydrogenases 2 which mediates the oxidation of estradiol to estrone, testosterone to androstenedione and androstendiol to dehydroepiandrosterone. The expression of 17β-hydroxysteroid dehydrogenases 1 and 2 alone and in combination has been shown to predict patient outcome, and inhibition of 17β-hydroxysteroid dehydrogenases 1 has been proposed to be a prime candidate for inhibition in patients who develop aromatase inhibitor resistance or in combination with aromatase inhibitors as a first line treatment. Here we review the status of inhibitors against 17β-hydroxysteroid dehydrogenases 1. In addition, we review the involvement of 17β-hydroxysteroid dehydrogenases 4, 5, 7, and 14 in breast cancer.

## INTRODUCTION

Sex steroid hormones such as estrogens and androgens are involved in the development and differentiation of several tissues and organs, including bone, cardiovascular, brain and gender-specific sites such as testis, prostate, endometrium, and ovaries. Steroids continuously assert their influence based on relative concentration and exposure time, which in turn is dependent on the circulating concentrations of the respective steroid, but also on local conversion.

**Figure 1:**
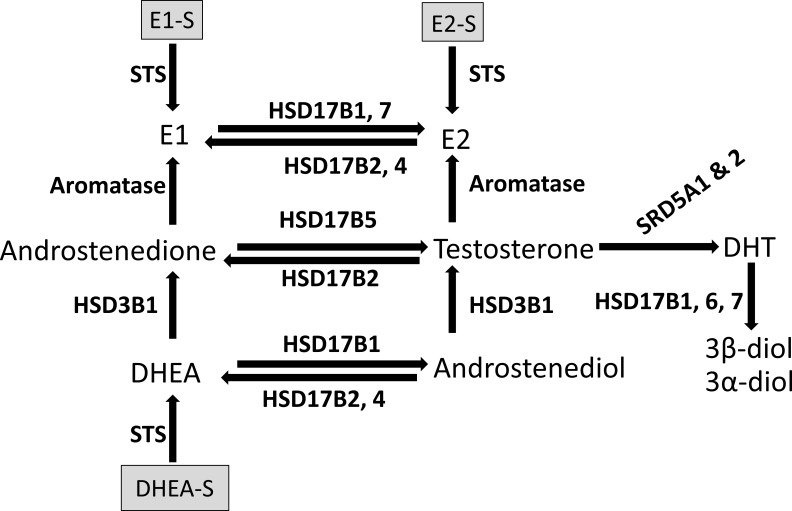
Schematic representation of the enzymatic conversion of sex steroids in breast tissue DHEA = dehydroepiandrosterone. DHEA-S = dehydroepiandrosterone-sulfate. DHT = dihydrotestosterone. E1 = estrone. E2 = estradiol. E1-S = estrone–sulfate. E2-S = estradiol-sulfate. HSD3B1 = hydroxysteroid 3 beta-1. HSD17B = hydroxysteroid 17-beta dehydrogenase. STS = steroid sulfatase. SRD5A1 & 2 = steroid 5 alpha-reductase 1 and 2.

The effect of sex steroids on breast tissue in genetic females is normally primarily mediated by estrogens, with estradiol being the most active estrogen. Estrogen signaling results in breast growth, and changes in estrogen exposure occur naturally in the different stages of life, such as puberty and pregnancy. The effect of androgens in breast tissue, with dihydrotestosterone (DHT) being the most potent, are in direct opposition to those of estrogens, mediating tissue homeostasis, and protection against proliferative signals and can lead to breast atrophy. Sufficient androgen concentrations prevent the formation of breasts, even in genetic females, in certain disorders such as adrenal tumors. The balance of estrogens and androgens thus determine the future of the breast in any individual, and uncontrolled estrogen signaling is the most widely accepted risk factor for breast cancer [1]. The primary site of estrogen production in premenopausal women is the ovaries, while most androgens are synthesized in the adrenal glands [2]. In postmenopausal women, the ovarian production of estrogens is greatly diminished, and adrenal androgens and sulfated estrogens become the primary circulating steroids. As a result of this shift, the primary source of active estrogen in any tissue becomes the product of local conversion. This conversion in the breast tissue is primarily mediated by a number of enzymes, including aromatase, steroid sulfatase (STS) and 17β-hydroxysteroid dehydrogenase (HSD17B) (Figure 1) [3–9]. The relative expression of the different enzymes, combined with the availability of substrates, mediates the balance and thus the final effect of the sex steroids in the local tissue.

## BREAST CANCER

Breast cancer is the malignant growth of cells in the breast tissue, most frequently the epithelium of the duct or lobule. During their lifetime, 10% of women will be diagnosed with breast cancer. Many breast cancers are steroid hormone dependent, and estrogen and androgen signaling have been shown to be the primary sex steroid hormones involved in regulating tumor growth and progression, with 70-80% of all breast cancers expressing estrogen receptor (ER)α [10, 11] and 60-80% expressing the androgen receptor (AR) [12, 13]. Estrogen signaling by ERα in breast cancer cells results in proliferation and survival signals while suppressing the expression of antiproliferative and apoptotic targets [14, 15]. Additionally, there is a second form of ER, known as ERβ (or ERβ1), which has growth inhibitory properties in breast cancer and can bind to ERα, forming heterodimers which have reduced transcription potential [15]. Further, ERβ splice variants ERβ2, ERβ3, ERβ4, and ERβ5, have reduced ligand binding capacity, and function through heterodimer formation with ERβ1 or ERα, modulating their activity and function. This topic is further discussed by Haldosen et al., and Sareddy et al., [16, 17]. The primary form of ER in healthy breast and most breast cancers is ERα, and as a result, most estrogen signaling is mediated through ERα signaling in breast cancer [15, 18]. Androgens, on the other hand, signal through AR. In ERα-positive tissues, such as healthy breast and ERα-positive breast cancer, androgens are reported to be primarily anti-proliferative and are associated with improved outcome [19–23]. The role of androgens in ERα-negative disease is more controversial, showing either improved or worsened patient outcome when expressed. This discrepancy likely depends on confounding factors such as the presence of AR splice variants, V-7 in particular [24, 25], the presence of HER2 [26], the presence of FOXA1 [27], variations in the grade of the cohorts studied, and differing AR protein cut-off values [23, 28, 29]. In ERα-negative disease, clinical trials evaluating the effect of antiandrogens in AR-positive patients are showing promising results from treatment with antiandrogens bicalutamide and enzalutamide [30]. This review will focus on the HSD17B family, which modulates 17β-hydroxysteroid activity through reduction or oxidation of the carbon at the 17^th^ position, and whose expression levels are frequently altered in breast cancer [6, 31–34].

## HSD17BS

The HSD17B family was first reported in the 50's when enzymes mediating conversation of 17β- hydroxysteroids (androgens and estrogens) in the placenta were discovered [34]. In the 90's the first members of the HSD17B family were cloned, sequenced and their function documented [3–7]. The enzymes of the HSD17B family are numbered in the order in which they were discovered. To date, 14 members have been identified, and with the exception of HSD17B5, which is an aldo-keto reductase (AKR), they are all part of the short-chain dehydrogenase/reductase (SDR) family. The HSD17Bs share a relatively low sequence homogeneity, approximately 20-30%. Despite this, there is a substantial overlap in enzymatic activity between family members, with HSD17B1, 3, 5, 7 and 12 catalyzing reduction and 2, 4 and 14 the oxidation of 17β-hydroxysteroids. The primary differences between the different reductive and oxidative members are the preferred substrate and their pattern of expression. Since the reduced forms of both androgens and estrogens (testosterone and estradiol (E2), respectively) have higher binding affinity to their respective receptors than their oxidized counterparts (androstenedione and estrone (E1) respectively), the oxidizing reaction is considered protective against the effects of sex hormones. In light of this, it is unsurprising that the enzymes which catalyze the oxidizing reactions are more widely expressed than the reductive counterparts, and are sometimes reduced or lost in cancer [3–7, 35–38]. The reducing forms of HSD17B enzymes are primarily expressed in the testis and ovaries but are also upregulated in some cancers [39–43].

## HSD17B1 AND 2

HSD17B1 was the first type of HSD17B enzyme discovered, the gene *HSD17B1* is localized to 17q11-q21 and encodes a 6 exon protein composed of 328 amino acids with a molecular mass of 34.95 kDa. The enzyme is expressed in the cytoplasm [6]. HSD17B1 is active as a homodimer composed of two subunits. The enzyme catalyzes reactions that increase the estrogenic activity of its ligands. The primary role of HSD17B1 is to mediate the reduction of E1 to E2, and HSD17B1 has been shown to be the most active enzyme in regards to E2 production [39]. HSD17B1 also catalyzes the reduction of *Dehydroepiandrosterone* (DHEA) to androstenediol, which has reduced androgenic and increased estrogenic activity [44, 45]. More recently, it has also been shown to metabolize DHT into 3β-diol and 3α-diol [46], both of which have much lower affinity for AR and increased affinity for ERβ and to some degree ERα compared to DHT [47–49]. Maintenance of low DHT concentration in the breast tissue is important for ERα-positive breast cancer since increased DHT concentrations will result in inhibition of proliferation [50, 51]. HSD17B1 is primarily expressed in the placenta and ovary [6], but it is also expressed at lower levels in breast epithelium [35, 36].

*HSD17B2* is localized to 16q24.1-q24.2 and encodes a 6 exon protein composed of 387 amino acids with a molecular mass of 42.785 kDa. The enzyme contains an endoplasmatic reticulum retention motif, which indicates this is a likely site for the protein to mediate its function [5]. HSD17B2 catalyzes the oxidation of E2 to E1, testosterone to androstenedione and androstenediol to DHEA [52]. HSD17B2 is expressed in placenta, lung, liver, pancreas, kidney, prostate, colon, small intestine, endometrium [6] and breast epithelial cells [35].

## ROLE OF HSD17B1 AND HSD17B2 IN BREAST CANCER

In the healthy breast, the oxidative reaction of estradiol catalyzed by HSD17B2 is preferred over the reductive reaction [35, 36]. *In vitro,* and *in vivo* studies using cell lines in rats and mice, as well as clinical studies have shown that the preferential reaction is reductive, and HSD17B1 expression has been found to be increased in breast cancer compared with unchanged tissue. This change is accompanied by increased E2 levels [53–57]. In postmenopausal patients, the circulating E1 is decreased, and the ratio of E2/E1 becomes higher in the tumor tissue. This is accompanied by increased *HSD17B1* mRNA expression levels, but no change in aromatase or sulfatase levels [58]. Using HSD17B1 expressing mice xenografts, Husen et al demonstrated that E1 induced tumor growth could be greatly inhibited by administration of HSD17B1 inhibitors [59]. A similar study was conducted where inhibition of HSD17B1 activity prevented the proliferation of breast cancer cells *in vitro*, and reduced tumor volume and E2 plasma concentration in human breast cancer cell lines grown *in vivo* using mice and rat models [57]. More recently, studies using breast cancer cells where HSD17B1 was downregulated also show a significant reduction in proliferation and lowered E2 concentrations, and accompanied by increased DHT levels, likely as a result of the loss of E1 to E2 and DHT to 3α/3β-diol conversion by HSD17B1 [39, 46, 60]. This reduced proliferation could be the result of DHT-mediated growth inhibition since the addition of E2 did not completely rescue the proliferation [60], and the role of DHT in reducing breast cancer cell proliferation has been previously reported [39, 46]. Finally, Aka et al. recently demonstrated an estrogen independent function of *HSD17B1*, where it favors an anti-apoptotic gene profile when expressed, which in estrogen-independent cells could reduce proliferation [61].

The expression of HSD17B2 in breast cancer is important in its capacity to oxidize E2 into E1 and protect the tissue from its activity, and HSD17B2 expression was shown to be reduced in breast cancer compared with benign tumors [56, 62]. Furthermore, *HSD17B2* mRNA expression has been shown to be inversely correlated to E2 levels in breast cancer [54] and to the majority of adverse clinical factors studied [63–65]. The role of HSD17B2 in ERα-negative breast cancer is likely different since its expression has been shown to be increased [53]. Recently, HSD17B2 expression has been shown to be significantly higher in invasive lobular carcinoma (ILC) than in invasive ductal carcinoma (IDC), and it was accompanied by reduced tumor size when expressed [66].

## THE CLINICAL RELEVANCE OF HSD17B1 AND HSD17B2

The clinical relevance of HSD17B1 has been highlighted in several patient cohorts. Oduwole et al show that in a primarily post-menopausal cohort, patients with tumors expressing HSD17B1 mRNA or protein had significantly shorter overall and disease-free survival than the other patients [40]. In a study conducted in our lab, two different post-menopausal cohorts showed that a high HSD17B1 to HSD17B2 ratio, as well as high HSD17B1 on its own was associated with worse prognosis and increased risk of recurrence in patients with ERα-positive tumors [65, 67]. Patients with high tumoral HSD17B2 expression or a high HSD17B2 to HSD17B1 ratio had improved prognosis on their own and was associated with reduced risk of recurrence in patients with ERα-positive tumors. Additionally, increased HSD17B1 expression was associated with increased risk of recurrence after 5 years [65, 67]. When analyzing copy number variation of the *HSD17B1* gene it was shown that increased copy number was correlated with decreased breast cancer survival [68]. The ratio of HSD17B1 to HSD17B2 has been shown to be a good indicator of tamoxifen treatment benefit, as post-menopausal patients with tumors expressing a high HSD17B1/HSD17B2 protein ratio have less benefit from tamoxifen treatment [69], likely as a result of increased E2 levels which can compete with tamoxifen, limiting its ability to prevent estrogen signaling [39, 52, 69]. Further, in ERα-positive pre-menopausal breast cancer patients who received tamoxifen treatment, low HSD17B1 expression was associated with reduced risk of recurrence [70].

## INHIBITORS OF HSD17B1 AND HSD17B2

Several authors have proposed the use of HSD17B1 inhibitors for breast cancer, either as a single treatment, conceivably once resistance to aromatase inhibitors has arisen, or in combination with other treatments [46, 71, 72]. The primary result of such inhibition would be the reduction of E2 levels and increased DHT levels in the tissues that express HSD17B1 [39, 44–46], and as such, side effects should be more limited than current anti-hormonal treatments due to the limited tissue expression of HSD17B1 in placenta, ovary [6] and breast epithelium [35, 36]. However, it is worth noting that different HSD17B enzymes, as well as compensatory mechanisms for the loss of the HSD17B1 mediated conversion of E1>E2, DHEA>androstenediol, and DHT>3β/3α-diol may result in variations in the systemic hormonal balance, beyond the effect in the breast tissue, following HSD17B1 inhibition. These changes would have to be evaluated during *in vivo* and clinical testing in order to validate the implications.

There are two primary forms of inhibitors available, steroidal and nonsteroidal [73, 74]. Several steroidal and non-steroidal compounds have been tested, and have shown to be able to reduce HSD17B1 activity *in vitro*, but the list of *in vivo* validated inhibitors is much shorter. Studies of the steroidal compound STX1040 on human breast cancer cells in mice and rat models showed that it reduced E1 induced tumor growth and E2 plasma concentration [57]. The non-steroidal compound B10720511 was shown to reduce tumor weight in mice by 60% when given in combination with E1 [59]. The compound PBRM [3-(2-bromoethyl)-16β-(m-carbamoylbenzyl)-17β-hydroxy-1,3,5(10)-estratriene] results in reduced T47D tumor burden in mice treated with E1 to levels which were similar to E1 untreated controls [75]. Despite a plethora of tested inhibitors, there is currently no clinically used HSD17B1 inhibitors, and more testing is needed to find suitable candidates.

## CONTROL OF EXPRESSION AND REGULATION OF HSD17B1 AND HSD17B2

The genetic aspects of HSD17B1 regulation are partially characterized, and HSD17B1 has been shown to have a promotor in the 5′ flanking region from -78 to +9, and a silencer element located -113 to -78. Further, the binding sites of transcription factors specificity protein (SP)1 and SP3 are present at -52 to -43, and regulate 30-60% of promotor activity. Additionally, activating protein (AP)2 binds at -62 to -53 and mediate a decrease in SP1 and SP3 binding, and a GATA3 binding site in the HSD17B1 silencer region was found to be associated with downregulated promotor activity [76]. The impact of HSD17B polymorphisms appears limited. A recent meta-analysis on the impact of HSD17B1 polymorphism rs605059 shows that it might confer genetic cancer susceptibility in Caucasians, but authors propose more studies are needed [77]. On the other hand, the SNP rs4445895_T was shown to be associated with lower intratumoral HSD17B2 mRNA levels and inversely correlated with E2 levels [78], indicating that HSD17B2 polymorphisms may have clinical relevance.

There have been several studies showing the impact of different therapeutic compounds on the HSD17B1 and HSD17B2 expression. It has been reported that progestins, used as a treatment for endometriosis or included in hormone replacement therapy can influence the oxidative and reductive capacity of tissues, and medrogestone, 20-dihydro-dydrogesterone, and nomegestrol acetate all reduce HSD17B1 activity by 35-51% in cell lines tested [36, 55]. In the strongly progesterone receptor (PgR) positive breast cancer cell line T-47D, progesterone, levonorgestrel, and medroxyprogesterone acetate were shown to up-regulate HSD17B1 and HSD17B5 expression and down-regulate HSD17B2 expression, with smaller effects seen on HSD17B1 expression in the moderately positive MCF7 [79]. Recently the progestin Dienogest was shown to down-regulate both HSD17B1 and aromatase expression in endometriosis patients, if this is also applicable in breast cancer remains to be seen [80]. In addition, HSD17B1 has long been known to be under the positive stimulatory influence of growth factors like insulin-like growth factors Types I and II and retinoic acid and immunological factors like interleukin 1 (IL-1), IL-6 and tumor necrosis factor α (TNFα) and it is possible that the cells of the immune system are an important source of the factors that modulate the expression and activity of HSD17B1 is breast tumors [53]. In postmenopausal ERα-positive breast cancer patients, the HSD17B1 expression was shown to be increased following steroidal aromatase inhibitor exemestane treatment. The authors hypothesized that this increase may be a response to estrogen depletion in an attempt by the breast tissue to increase local estrogen concentration using estrogen producing pathways other than aromatase [81]. Similar findings were made in lung cancer cell lines A549 and LK87 where aromatase inhibitor treatment resulted in increased HSD17B1 expression [82]. In breast cancer cell line T-47D, which is ERα- and AR-positive, treatment with the aromatase inhibitor exemestane was shown to result in increased HSD17B2 expression, a change which was associated with increased DHT expression. Both DHT and exemestane directly upregulated HSD17B2 expression in an AR-dependent manner and this effect was counteracted by E2 [83]. Furthermore, treatment with inhibitors of 5alpha-reductase type I and type II in prostate cancer cell lines resulted in increased HSD17B1 [84], suggesting a role of DHT in up-regulating HSD17B1 expression in prostate cancer cells. HSD17B1 has been shown to be under the regulation of microRNAs 210 and 518c in placental cells [85] and microRNAs-10b, 145, 342, 17, 26a and 106b have been predicted to interact with HSD17B1 and HSD17B2 in breast cancer [86]. As of the writing of this review, no publications experimentally examining the role of miRNAs in regard to HSD17B1 and 2 in breast cancer has been published.

## HSD17B1 AND HSD17B2 IN OTHER FORMS OF CANCER

Besides their role in breast cancer, which is relatively well-documented, HSD17B1 and HSD17B2 are also involved in several other forms of cancer. In this section colon cancer, lung cancer and prostate cancer will be discussed.

In the healthy colon, HSD17B2 is normally expressed in the epithelial cells of the colon lumen, and to a lesser extent in the crypt epithelium [87]. HSD17B2 and ERβ are widely expressed at relatively high levels, while HSD17B1 and ERα are weakly expressed or not expressed at all. Aromatase expression is relatively low in healthy colon tissue, and is unchanged in colon cancer patients, suggesting that it is not involved in colon cancer pathogenesis [88]. Further, in the colon, E1 but not E2 has been shown to inhibit proliferation [87]. In the healthy colon, the proliferating cells of the colon crypts normally express no HSD17B2 and gain HSD17B2 as they differentiate and migrate towards the colon lumen [64]. Colon cancer reverts to the proliferative phenotype of the crypt, as there is a reduction in the oxidative activity compared with matched controls, accompanied by the loss of HSD17B2 and 4 expression. As a result, the E2 to E1 ratio is increased in colon cancer. This change is accompanied by increased proliferation [87, 89]. Additionally, ERβ is reduced in colon cancer, which has been shown to be a prognostic marker for worse prognosis [88]. It has been shown that distal colon carcinoma may not mimic proximal colon cancer and that HSD17B2 expression may be an independent factor of poor prognosis in distal colon cancer [64, 90].

In non-small cell lung cancer (NSCLC) the expression of HSD17B1 is increased as compared with healthy tissue [91]. Moreover, HSD17B1 is correlated to higher stage and increased E2 concentration, meanwhile HSD17B2 expression is correlated to lower stage and to increased E1 concentration [82]. NSCLC cell lines capable of catalyzing the E1 to E2 conversion were shown to be HSD17B1 positive, which supports a role of HSD17B1 as mediator of this conversion [92]. Further, HSD17B1 is an independent negative prognostic factor [82, 91].

The healthy prostate expresses HSD17B2 and the amount of HSD17B2 expression is reduced in prostatic carcinoma compared to benign hyperplasia [93]. In prostate cancer, HSD17B2 SNPs rs4243229 and rs7201637 were associated with progression in both Caucasian and Taiwanese cohorts studied [94]. Additionally, SNPs rs1364287, rs2955162, rs1119933, rs9934209 were also associated with prognosis in terms of progression in a Caucasian cohort [95]. No expression of HSD17B1 has been reported in prostate cancer, and the primary reductive HSD17B in prostate cancer is HSD17B5. Moreover, higher HSD17B5 expression is correlated with advanced stage of disease [42, 96, 97]

## OTHER HSD17B ENZYMES

While this review focused on the roles of HSD17B1 and HSD17B2, in breast cancer, this section will briefly describe other family members of note, with a focus on their role in breast cancer.

HSD17B4 is expressed in virtually all human tissues, similar to HSD17B2. It mediates the transformation of E2 to E1 and androstenediol to DHEA when expressed [3], however, its activity is reported to be much lower than of HSD17B2 and its importance in steroid formation in the human remains to be established [98].

HSD17B5 is expressed in healthy ovarian tissue and healthy breast ductal epithelium. It is overexpressed in several forms of cancer, including breast cancer [40, 41], prostate cancer [42] and ovarian cancer [43]. This enzyme catalyzes the conversion of testosterone from androstenedione and facilitates the inactivation of DHT and progesterone [4, 40]. High HSD17B5 expression has been shown to be correlated with worse prognosis [40] and increased risk for late relapse in ERα-positive patients who were recurrence-free after 5 years [99]. Further, HSD17B5 expression is correlated with 5α-reductase expression in breast cancer [41].

HSD17B6 is primarily involved in the prostate, mediating the conversion of DHT to 3α and 3β-diol [100]. This is supported by the fact that HSD17B6 and ERβ are often colocalized in prostate tissue [101]. In triple negative breast cancer, it was shown to be associated with improved outcome, likely by promoting ERβ signaling [102].

HSD17B7 is expressed in the ovary, placenta, breast tissue, testis, liver, and brain [37]. It catalyzes the conversation of E1 to E2 and reduction of DHT [7, 39]. Knocking down HSD17B7 expression in breast cancer cell lines resulted in a marked reduction in proliferation, suggesting it may be a potential target for treatment of ERα-positive breast cancer [39].

HSD17B14, being primarily oxidative in nature, is expressed in the endometrium, ovaries, breast, testis, GI, kidney and retina [38]. HSD17B14 uses NAD as a cofactor and was shown to catalyze the conversion of E2 to E1 and androstenediol to DHEA [32, 103]. More recent experiments have put this into question since very low steroid converting activity was measured compared to cells expressing HSD17B2 [38]. It is expressed in breast cancer patients. High HSD17B14 mRNA expression can predict improved recurrence-free survival and breast cancer-specific survival [99]. Further, HSD17B14 has been shown to predict the effect of tamoxifen treatment in terms of recurrence-free survival in ERα-positive lymph node negative breast cancer patients [104].

## CONCLUDING REMARKS

Steroid hormones are pivotal in determining the future of tissues exposed to them, and HSD17Bs, especially 1 and 2, are important components in mediating the local concentrations of steroids in tissues where they are expressed. The evidence is mounting that they are involved in several forms of cancer, and the expression pattern of HSD17Bs differs greatly in cancer as opposed to healthy tissue. Their role in breast cancer is highlighted by the frequently lost expression of the oxidative protective HSD17B2 and 4, combined with increased expression of HSD17B1, 5 and 7. As a result, the tissue is exposed to increased concentrations of proliferative estrogens and reduced anti-proliferative androgens, resulting in disease progression. The implications for HSD17B1 as a treatment target have been known for a while, but the successful development of an inhibitor which can be brought into clinical trial is unfortunately not yet achieved. Future analysis of the cause of the changes in HSD17B expression between disease and health, could provide an alternate avenue of treatment, if it would open the possibility restoring a tissue protective HSD17B pattern in disease tissue. Further, analysis of the expression of HSD17Bs has been shown to be predictive of treatment and prognostic in several cancers, and as such would be a candidate for routine examination in a clinical setting.

## Abbreviations

AKR: aldo-keto reductase
AP: activating protein
AR: androgen receptor
DHT: dihydrotestosterone
DHEA: Dehydroepiandrosterone
E1: estrone
E2: estradiol
ER: estrogen receptor
HSD17B: 17β-hydroxysteroid dehydrogenase
IDC: invasive ductal carcinoma
ILC: invasive lobular carcinoma
IL-1: interleukin 1
NAD: Nicotinamide adenine dinucleotide
PBRM: 3-(2-bromoethyl)-16β-(m-carbamoylbenzyl)-17β-hydroxy-1,3,5(10)-estratriene
PgR: progesterone receptor
SDR: short-chain dehydrogenase/reductase
SP: specificity protein
STS: steroid sulfatase; TNFα
